# Corrigendum to “New Issues in the Management of Osteoporosis”

**DOI:** 10.1155/2017/7174960

**Published:** 2017-11-22

**Authors:** Joonas Sirola, Manuel Diaz Curiel, Risto Honkanen, Jun Iwamoto

**Affiliations:** ^1^Bone and Cartilage Research Unit, University of Kuopio, 70211 Kuopio, Finland; ^2^Metabolic Bone Diseases, Department of Internal Medicine, Autonomous University of Madrid and Jimenez Diaz Foundation, 28049 Madrid, Spain; ^3^Institute for Integrated Sports Medicine, School of Medicine, Keio University, Tokyo 160-8582, Japan

In the article titled “New Issues in the Management of Osteoporosis” [1], there was an error regarding the FRAX® tool, which should be clarified as follows.

The article notes, “It introduces and validates the Mikkeli Osteoporosis Index (MOI) and compares its performance to WHO FRAX tool in separate Finnish cohorts of postmenopausal women. The performance of MOI was found to be comparable to that of World Health Organization (WHO) FRAX-tool in identification of intervention thresholds.” However, the World Health Organization (WHO) did not develop, test, or endorse the FRAX tool or its recommendations [2]. The metabolic bone disease unit at the University of Sheffield that developed FRAX was a WHO Collaborating Centre from 1991 to 2010, but treatment guidelines must undergo a formal process before they can be endorsed by the WHO.
